# Ambiguity in robotic surgical instruction: lessons from remote and in-person simulation

**DOI:** 10.1007/s10459-024-10408-1

**Published:** 2025-01-17

**Authors:** Riley Brian, Laura Sterponi, Alyssa Murillo, Daniel Oh, Hueylan Chern, Elliott Silverman, Patricia O’Sullivan

**Affiliations:** 1 Department of Surgery, University of California San Francisco, 513 Parnassus Avenue S-321, San Francisco, CA 94143 USA; 2 Berkeley School of Education, University of California Berkeley, Berkeley, CA USA; 3 Department of Surgery, University of Southern California, Los Angeles, CA USA; 4 Intuitive Surgical, Sunnyvale, CA USA

**Keywords:** Robotic surgery, Surgical instruction, Communication, Discourse analysis, Ambiguity

## Abstract

The rise of robotic surgery has been accompanied by numerous educational challenges as surgeons and trainees learn skills unique to the robotic platform. Remote instruction is a solution to provide surgeons ongoing education when in-person teaching is not feasible. However, surgical instruction faces challenges from unclear communication. We aimed to describe, examine, and compare ambiguities in remote and in-person robotic instruction. We designed a simulation scenario in which a standardized learner performed tasks in robotic surgery while making pre-scripted errors. Instructors provided remote or in-person instruction to the standardized learner. We applied tools from discourse analysis to transcribe sessions, identify instructional instances, classify ambiguities, and select passages for further review. We used tests of proportions to compare ambiguities between the settings. We conducted four simulation sessions, including two remote and two in-person sessions, and identified 206 instructional instances. Within these, we found 964 occurrences of three common semantic ambiguities, or ambiguities arising from words alone. Instructors used visual tools – thus employing multimodality – to clarify semantic ambiguities in 32% of instructional instances. We identified a similar degree of referential ambiguity, or ambiguity for which context from multimodality did not provide clarifying information, during remote (60%) and in-person (48%) instructional instances (*p* = 0.08). We described, examined, and compared ambiguities in remote and in-person instruction for simulated robotic surgery. Based on the high prevalence of ambiguity in both settings, we recommend that robotic instructors decrease referential ambiguity. To do so, instructors can reduce semantic ambiguity, harness multimodality, or both.

## Introduction

Clear communication is essential throughout health care, with negative patient and learner outcomes a possible result of poor communication (Belyansky et al., 2011; Foronda et al., 2016; Goodboy et al., 2023). In the operating room, specifically, communication facilitates safe patient care and also plays a central role in intra-operative instruction (Chatterton et al., 2023). However, the landscape of operating rooms has changed recently with a dramatic rise in robotic surgery across general surgery, thoracic surgery, urology, and obstetrics-gynecology (Sheetz et al., 2020). This has been accompanied by numerous challenges as surgeons and trainees learn the cognitive, technical, and instructional skills unique to the robotic platform (Green et al., 2019; Lane, 2018; Muaddi et al., 2021).

In traditional open surgery, the sterile operative team stands in close proximity at the operating room table and communicates face-to-face orally, with gestures, and by touch. In robotic surgery, communication transforms relative to open surgery due to physical separation among team members, workflow changes with less need for assistance, noise from the robotic platform, and unique visual aids (Cristofari et al., 2022; Siu et al., 2010; Wong et al., 2023). Such visual aids in robotic surgery include animated pointer hands, static image illustration, and *telestration*, a tool that involves drawing on the operative screen through a video monitor close to the patient’s bedside. The operating robotic surgeon can view these visual aids on the surgeon console. These many differences in robotic surgery upend the traditional operative communication strategies used for instruction.

Access to high-quality robotic surgical education is not universal across the country or world; as such, instructors have implemented remote instruction as a potential solution for surgeons with limited robotic surgical experience to receive expert guidance during an operation through telepresence (Santomauro et al., 2013). Instructors teach remotely in a number of forms, but the most common approach involves an expert watching the operation from a computer and providing audio feedback to a surgeon headset and visual support with an animated pointer (Butt & Augestad, 2021). Surgeons have reported multiple benefits of remote instruction, including its efficiency and cost savings (Artsen et al., 2021; Brook et al., 2019; Hung et al., 2018). Unfortunately, multiple obstacles have prevented the widespread adoption of remote instruction. Most prior work has described the technological, legal, ethical, and safety challenges (Hung et al., 2018). However, communication – already altered in robotic compared to open surgery – further changes in the setting of remote instruction. In remote instruction, the expert surgeon and learning surgeon are geographically separated and have more limited tools to understand each other.

Tools to enable instruction, whether remote or in-person, take *multimodal* forms. Indeed, the social semiotic theory of multimodality highlights the potential of non-verbal communication (Kress, 2009; Wyatt-Smith & Cumming, 2009). There are several key premises of multimodality, including that meaning is made using different semiotic (sign and gesture-based) resources and that we must attend to all such resources to truly understand communication (Jewitt et al., 2016). Multimodality can be employed to reduce *semantic ambiguity*, or ambiguity resulting purely from the words used to communicate (Wyatt-Smith & Cumming, 2009). Semantic ambiguities, without nonverbal context, create *referential ambiguity* (Box 1). Referential ambiguity comprises ambiguities for which context does not provide sufficient information for a listener to infer an appropriate interpretation about the meanings (or references) of words (Macagno & Bigi, 2018). Thus, referential ambiguity disrupts clear communication. Multimodality plays centrally in the relationship between semantic and referential ambiguity. By providing additional context, multimodality can obviate the ambiguity of words alone (semantic ambiguity) to help those communicating avoid referential ambiguity.

**Box 1:** This non-medical example contrasts *semantic ambiguity* and *referential ambiguity*. It also contrasts the three common semantic ambiguities highlighted in this study: *spatial deixis*, *anaphora*, and *directional frames of reference*.

**Example of Types of Ambiguity**
If a driver were asking a pedestrian for directions and the pedestrian said, “it’s over there, to the left,” such advice would be semantically ambiguous in multiple ways. First, “over there” is spatially deictic, meaning the pedestrian is referring to a location (“there”) without specially naming that location. Second, “it” represents anaphora, as accurate interpretation of “it” requires understanding of an antecedent. Third, “to the left” is a directional frame of reference, in that interpretation of “to the left” may vary depending on how the pedestrian and driver are looking. Semantic ambiguity alone does not necessarily lead to misunderstanding. If the pedestrian had pointed while speaking, pointing – as a visual tool – would represent a type of multimodality. The use of multimodality in this setting may allow the driver to understand the instructions. Thus, verbal instructions accompanied by multimodality can reduce referential ambiguity by providing visual context to ground instruction.

Prior authors have identified large numbers of semantic ambiguities in open and laparoscopic surgery, though these authors have also confirmed the importance of multimodal tools to supplement speech and clarify communication (Emmerton-Coughlin et al., 2017; Koschmann et al., 2011; Liu et al., 2021; Mondada, 2014). These studies have solely investigated ambiguity in non-robotic settings that lack the unique physical and communicational aspects of robotic surgery. It is unknown how the altered communication dynamics in robotic remote and in-person instruction affect semantic or referential ambiguity. Indeed, previous work assessing robotic surgical education has specifically called for more investigation into communication with regard to instruction in the robotic operating room (Green et al., 2020). There is a gap in our understanding of how communicational ambiguities manifest in robotic surgical instruction. Given the rise in robotic surgery as a key modality and the importance of remote instruction in this rise, better recognition of ambiguity during remote and in-person robotic surgical instruction may enable improved learning and safer patient care. As such, we aimed primarily to describe and examine ambiguities in robotic surgical instruction, and secondarily to compare ambiguities between remote and in-person robotic instruction.

## Methods

### Study design

We designed a study to describe ambiguities in robotic surgical instruction, examine the ambiguities, and compare the ambiguities between remote and in-person teaching. To do so, we created a simulation scenario based on the Fundamentals of Robotic Surgery (FRS). FRS is a curriculum with validity evidence to assess the basic skills required of surgeons for the performance of robotic operations (Satava et al., 2020). We reviewed and applied known error taxonomies to create a scripted set of errors (Table 1) to be performed by a standardized learner while completing the FRS tasks (D’Angelo et al., 2015; Hutchinson et al., 2022; Rasmussen, 1982). The standardized learner was a trained actor who practiced to perform tasks in a pre-set fashion following the scripted set of errors (Boillat et al., 2012). We chose to use a standardized learner to reduce variability in learner performance. We then recruited senior trainees (post-graduate year 4 and above) and faculty members by email to participate as instructors. We assigned these instructors to provide remote or in-person instruction to the standardized learner as he performed the FRS tasks with the scripted set of errors. We recorded the audio and video of all sessions. Following the sessions, we manually transcribed each session using standard notation (Table 2).

**Table 1:** Tasks performed during the simulation sessions and the scripted errors produced during each task by the standardized learner

Task Name	Task Description	Scripted Errors
Ring Tower Transfer	Learner removes a ring from a curled wire and moves the ring to a different curled wire; the learner then repeats this with a second ring	Learner collides with a wire and with another instrument while removing the ring from another wire
Knot Tying	Learner ties a knot	Learner moves instruments out of the camera’s view while tying
Railroad Track	Learner sutures between dots to close a horizontal incision and then ties a knot	Learner puts excessive tension on the incision by not following the needle’s curvature and by failing to pull the suture through with each stitch
Fourth Arm Cutting	Learner stretches a length of silicone material and cuts at three designated points by using all of the robotic arms	Learner inappropriately leaves one of the robotic arms in the way of the other arms
Cloverleaf Dissection	Learner dissects a cloverleaf out from underlying material using scissors	Learner fails to adjust the robotic arms to provide tension and optimize the cutting angle
Vessel Energy Dissection	Learner dissects out a blood vessel and then ligates the vessel with bipolar energy	Learner applies an inappropriate type of energy when attempting to ligate the vessel

**Table 2:** Transcription notation used during data review and analysis as represented in Figs. 3 and 4

Notation	Definition
.	A period indicates a falling or final intonation
,	A comma indicates a continuing intonation
:	A colon indicates prolongation of the preceding sound, with the number of colons correlating with the length of prolongation
-	A hyphen indicates a cut-off word
word	Underlining indicates emphasis
> <	“Greater than” and “less than” signs in this configuration indicate that the contained speech is compressed
( )	Numbers within a single set of parentheses denote pauses in seconds
(.)	A period within a single set of parentheses indicates an audible pause too short to measure, generally 0.2 s or less
(( ))	Double parentheses indicate actions

Within transcripts, we identified *instructional instances*. We defined an instructional instance as comprising the speech and actions between instructor and learner about one discrete instructional topic. Next, we identified semantic ambiguities. Two authors coded all semantic ambiguities and reconciled codes. Finally, we reviewed every use of multimodality throughout the transcripts. After these analyses, all authors discussed ambiguities within transcripts and deliberated on recommendations based on the data.

### Study setting and equipment

We conducted this study at the University of California San Francisco (UCSF). UCSF is a large academic medical center with multiple clinical sites. We used a dedicated single-console robotic da Vinci Surgical System (DVSS) loaned to UCSF for education (Intuitive Surgical, Sunnyvale, CA). For remote instruction, we attached DVSS to an Intuitive Hub and connected the Hub to DVSS. The instructor then provided guidance from a laptop computer in another room to the standardized learner through Intuitive Telepresence. In addition to two-way verbal communication, Intuitive Telepresence allows the instructor to visualize the endoscope view, an over-the-bed view, and a wide-angle view. It also permits the instructor to display and move an animated pointer in a smaller screen on the surgeon console through the DVSS Tilepro™ function. Finally, it allows an instructor to illustrate a static image for the surgeon to view. For in-person instruction, the instructor and standardized learner were present in the same room during the simulated case. In these sessions, instructors had the ability to talk directly to the standardized learner and use telestration.

### Data analysis

We approached this study using tools from *discourse analysis*. Discourse analysis, as defined by Hatch (1992), is the “study of the language of communication – spoken or written.” Discourse analysis differs from traditional linguistic approaches, which look at language isolated from use rather than language in action (Goodwin, 2000). Discourse analysis is a powerful tool in understanding communication in instructional settings (Mehan, 1979; Waring, 2008). Importantly, understanding discourse requires making sense of the context beyond words including by understanding multimodality (Goodwin, 2000). Through a microanalytic lens, discourse analysis provides a tool to understand contextualized utterances (Schiffrin, 1994). This study applied principles from discourse analysis to analyze verbal and nonverbal language in remote and in-person robotic surgical instruction. We selected excerpts for this report using standard methods from discourse analysis (Gill, 1996; Potter & Wetherell, 1994). We paired these excerpts with images that appropriately contextualized the excerpts within the relevant discourse from the simulated cases.

We focused on three common semantic ambiguities in the transcripts: *deixis*, *anaphora*, and *directional frames of reference*. Deictic expressions include references to a location or time that link to the context in which they are used and without specifically naming that location or time; we focused on spatial and temporal deixis (Horn & Ward, 2004). Anaphora is closely linked to deixis, but comprises expressions for which accurate interpretation requires understanding of an antecedent (Huang, 2006). Directional frames of reference are expressions for which interpretation varies based on the physical relationships of objects and those speaking (Clementini, 2013). We compared instructional instances when multimodality was and was not used. We determined referential ambiguity to be present in an instructional instance if that instance included semantic ambiguity but did not include any use of multimodality.

We collected descriptive statistics about the number of instructional instances, the prevalence of the three common semantic ambiguities, and the use of multimodality. We compared the proportion of instructional instances with each of the three ambiguities between remote and in-person instruction using tests of proportions. We also compared the proportion of instructional instances with the use of multimodality between remote and in-person instruction using a test of proportions. Finally, we compared the proportion of instructional instances with referential ambiguity between remote and in-person instruction using a test of proportions. We used Stata/IC 16.1 for Mac for statistical analyses.

### Ethical considerations

Our Institutional Review Board determined this study to be exempt from review (IRB22-36383).

## Results

We conducted four simulation sessions, including two remote and two in-person sessions, resulting in 206 instructional instances. Four instructors participated in these sessions: one senior trainee and one faculty member participated as instructors in the remote sessions, and a different senior trainee and faculty member participated as instructors in the in-person sessions.

**Fig. 1:**
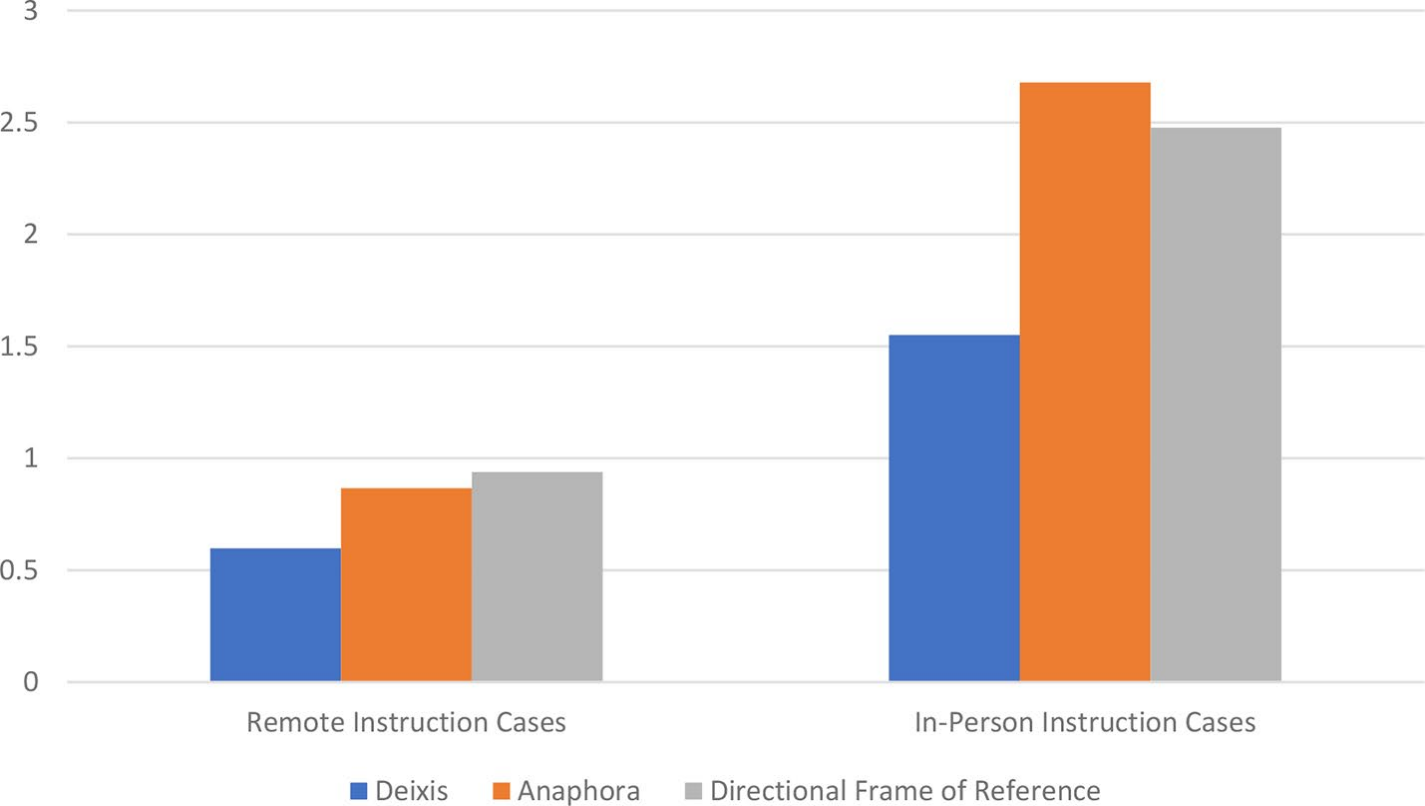
Average semantic ambiguities per instructional instance in the remote and in-person simulated robotic cases. All three semantic ambiguities were more common during in-person instruction than during remote instruction (*p* < 0.001 for all)

We first assessed semantic ambiguities. Among the 206 instructional instances, 86% contained one or more of the semantic ambiguities of deixis, anaphora, and directional frames of references (see Table 3 for examples). We found a total of 964 of the three semantic ambiguities. This equated to an average of 4.7 semantic ambiguities per instructional instance or 7.5 semantic ambiguities per minute. We identified 227 instances of deixis, 376 instances of anaphora, and 361 instances of directional frames of reference. Inter-rater agreement with regard to semantic ambiguities was 98.7%. All three semantic ambiguities were more common during in-person sessions (6.7 ambiguities per instructional instance) than during remote sessions (2.4 ambiguities per instructional instance) (*p* < 0.001 for all) (Fig. 1).

**Table 3:** Definitions, prevalence, examples, and alternatives for three common semantic ambiguities in the remote and in-person robotic simulation sessions

	Definition	Examples from Transcripts	Explanation of Example	Less Ambiguous Alternatives
Deixis *n* = 227	Expression referring to a location or time without specifically naming the location or time	“Pull right here”	“Right here” refers to a location that is not specifically named in the expression	“Pull right here” (with circle around area to pull) “Pull the tail of the suture”
		“You’re hitting the wire there”	“There” refers to a location that is not specifically named in the expression	“You’re hitting the wire there” (with arrow pointing to area of contact) “You’re hitting the wire with your needle driver”
Anaphora *n* = 376	Expression for which accurate interpretation requires understanding of an antecedent	“Grab this and lift it”	“This” and “it” refer to antecedents that may or may not have been previously stated	“Grab this and lift it” (with circle around area to grab and arrow pointing towards direction in which to lift) “Grab the free edge of the puzzle piece and lift superiorly”
		“Re-thread it through the other”	“It” and “other” refer to antecedents that may or may not have been previously stated	“Re-thread it through the other” (with circles around the object to re-thread and the object to be re-threaded) “Re-thread the suture through the wire loop”
Directional Frames of Reference *n* = 361	Expression for which interpretation varies based on the physical space of objects and those speaking	“Turn the camera towards the left”	“The left” can be interpreted differently based on the speaker’s, listener’s, or patient’s frame of reference	“Turn the camera towards the left” (with arrow pointing to the area of interest) “Turn the camera towards the patient’s left”
		“Spread behind there”	“Behind” can be interpreted differently based on the speaker’s, listener’s, or patient’s frame of reference	“Spread behind there” (with circle indicating where to spread) “Spread posterior to the blood vessel”

**Fig. 2:**
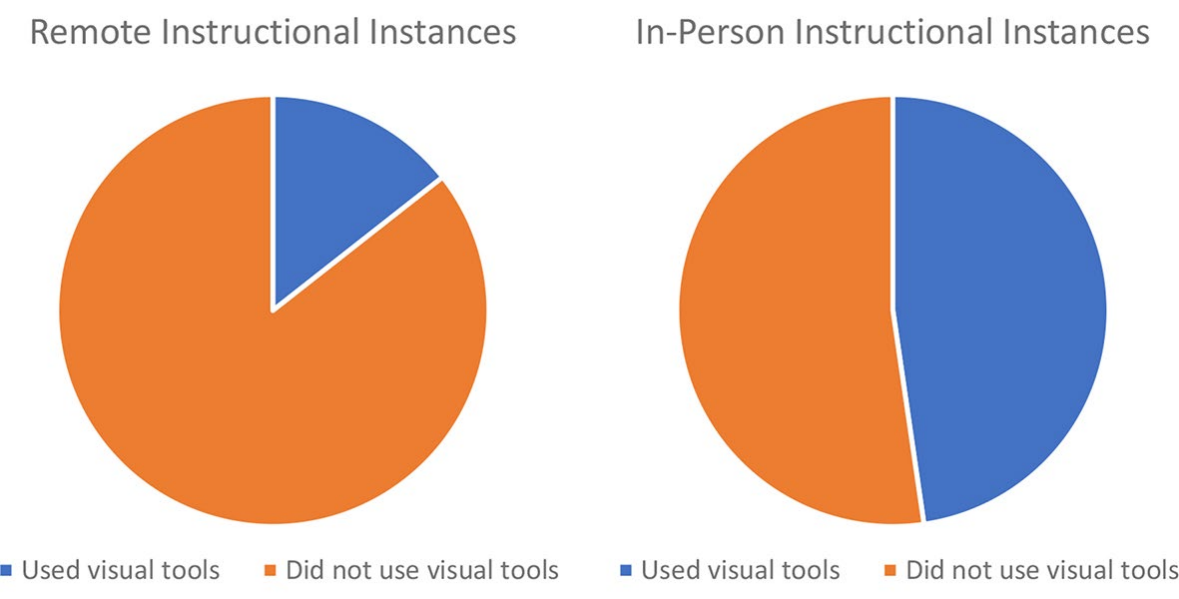
Instructors used visual tools less frequently during instructional instances in the remote (left) compared to the in-person (right) simulated robotic cases (*p* < 0.001)

Next, we looked at the use of multimodality. Instructors used the visual tools of pointing (during remote cases) and telestration (during in-person cases) in 32% of instructional instances, with significantly greater use during in-person (48% of instructional instances) than during remote (14% of instructional instances) instruction (*p* < 0.001) (Fig. 2). Remote instructors did not use the visual tool of static image illustration.

**Fig. 3:**
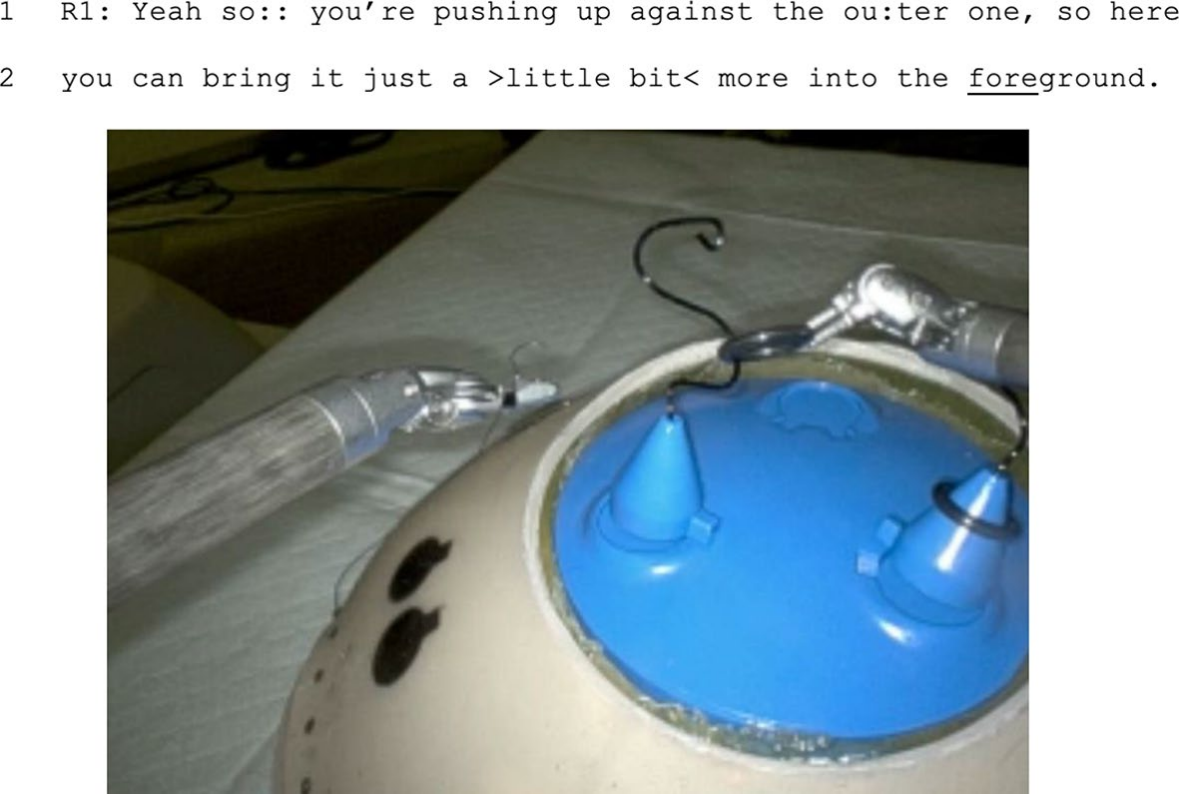
In many instances, instructors used semantically ambiguous expressions, including anaphora like “the outer one” or “it” without visual tools. In these cases, there was no multimodality that reduced referential ambiguity

**Fig. 4:**
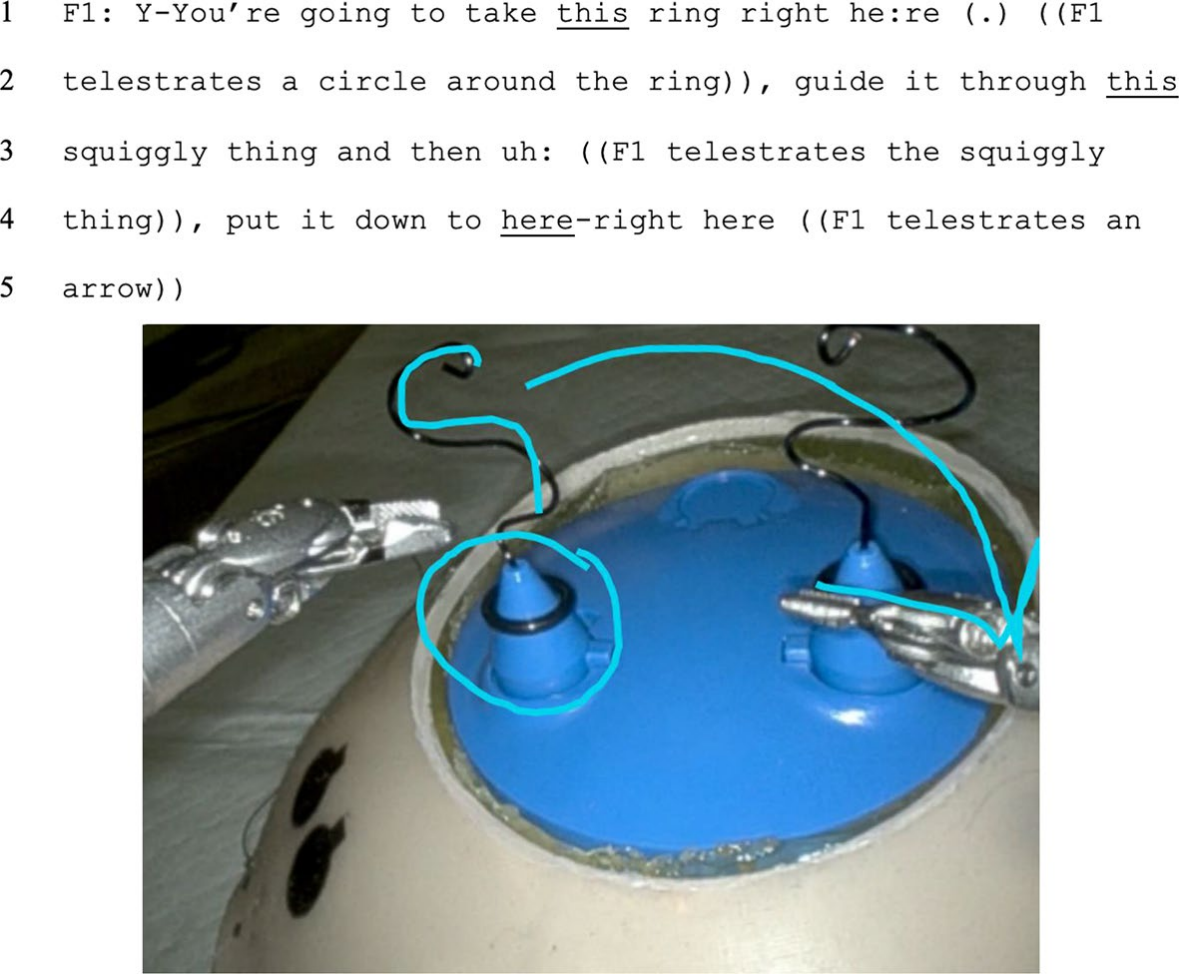
Visual tools like telestration (i.e., drawing on the operative screen as presented in light blue overlay) allowed the instructor to provide context to semantic ambiguities. While “right here” is spatially deictic and “it” and “thing” represent anaphora, telestration obviated the referential ambiguity associated with these phenomena

Finally, we assessed referential ambiguity. In 53% of instructional instances, instructors used semantically ambiguous instruction without multimodality, thus creating referential ambiguity (for example, as in Fig. 3). The amount of referential ambiguity was similar during remote (60%) and in-person (48%) instructional instances (*p* = 0.08). Instructional instances without referential ambiguity included both those instances without semantic ambiguity and those instances in which multimodality eliminated ambiguity (for example, as in Fig. 4).

## Discussion

We identified, examined, and contrasted ambiguities in remote and in-person instruction during simulated robotic surgery cases. Overall, a high prevalence of ambiguity manifested through the simulated cases. Although we found more semantic ambiguities per instructional instance during in-person instruction, we also found greater use of multimodality during in-person instruction, highlighting the difference between semantic and referential ambiguity. This distinction emphasizes the importance of mobilizing all available multimodal resources to effectively instruct and avoid referential ambiguity. In other words, while semantic ambiguity was significantly more prevalent during in-person instruction, the greater use of multimodality lent to a similar degree of referential ambiguity in remote and in-person robotic instruction. Even in the context of multimodality, however, we found referential ambiguity in the majority of instructional instances.

Given the prevalence of referential ambiguity during instruction, we recommend that those teaching robotic surgery work to reduce referential ambiguity through the use of semantic precision, multimodality, or both. Semantic precision requires avoiding ambiguities like deixis, anaphora, and directional frames of reference. The first step towards semantic precision is simple recognition of semantically ambiguous expressions as confusing for learners, who may lack the background and experience to infer appropriate meanings. Some such expressions (e.g., “to the right”) can be clarified through a prebriefing, in which instructors and learners agree on certain communication standards at the start of a case. For example, an instructor and learner may decide that all spatial directions will be based on the patient’s frame of reference. Other phrases (e.g., “it”) cannot be easily clarified through prebriefing and instructors must work to avoid such phrases to attain semantic precision. We also recommend that instructors harness multimodality whenever feasible. While the affordances of different visual tools vary, these tools may permit learners to see what the instructor sees, thus reducing referential ambiguity. Such visual aids can be effective even when simple, including with basic arrows and circles to provide visual references to accompany oral instruction. Finally, instructors can combine semantic precision and multimodality to describe clearly in words while illustrating visually. All three methods to reduce referential ambiguity – semantic precision, multimodality, or both – promote clear instruction.

The importance of reducing ambiguity and promoting clear instruction can be understood with a framework of *cognitive load theory*, which is a central idea in the understanding of operative and procedural education (Howie et al., 2023; Sweller, 1988). Originally outlined by Sweller in 1988, cognitive load theory has more recently been described as the idea that “an individual only has a limited volume of cognitive resource and that exceeding this volume resource demand beyond an individual’s capacity can compromise learning and the successful execution of a task” (Howie et al., 2023). One key contributor to cognitive load is *extraneous load*, which results from non-task related factors, including poor communication (Howie et al., 2023; Young et al., 2014). Multiple studies in the surgical education literature have focused on measuring and reducing cognitive load (Dias et al., 2018; Tokuno et al., 2023). One method to decrease cognitive load is to reduce extraneous load. Suboptimal communication – including through ambiguity – contributes to extraneous cognitive load, which may impair performance and learning (Anton et al., 2021; Weber et al., 2018; Young et al., 2014). Authors have suggested a number of instructor-focused interventions to reduce extraneous cognitive load in procedures (Sewell et al., 2017, 2019; Young et al., 2014). These suggestions have included avoiding distracting information, ensuring supervisor engagement, optimizing instructional pace, and of particular relevance: communicating with clarity (Greer et al., 2022; Young et al., 2014). The high prevalence of referential ambiguity identified in our study suggests a lack of clear communication during instruction. By reducing referential ambiguity, instructors may decrease learners’ extraneous cognitive load, thus enabling cognitive bandwidth for improved task performance and learning.

Prior studies have identified unique constraints and affordances of communication both more broadly in surgery and specific to robotic surgery. Several reported communication-related challenges overlap with those identified in this study. Chatterton et al. (2023) generated a list of “twelve tips” to optimize intra-operative learning, some of which relate to communication and effective intra-operative feedback (e.g., “use effective communication for both learning and patient safety”). In another study of medical students’ preferences for surgeon communication, clarity topped the list as the most important trait (Goodboy et al., 2023). In robotic surgery, specifically, Greenberg et al. conducted a small simulation-based study with medical students learning robotic surgery and noted issues with unclear language and hierarchy (Greenberg et al., 2022). Green and colleagues (2020) also studied simulated robotic surgical communication and similarly found communication challenges, particularly related to language precision. These studies are further supplemented by the instructional and communication literature from non-surgical fields (Wharton & Rossi, 2015). This study adds to the above work through its focused approach on semantic and referential ambiguity in remote and in-person robotic instruction.

Furthermore, from a methodological standpoint this study highlights the applications of microanalytic discourse analysis to surgical instruction. While limited prior work has assessed language and discourse in surgery, discourse analysis remains relatively unused in surgical education (Emmerton-Coughlin et al., 2017; Koschmann et al., 2011; Liu et al., 2021; Mondada, 2014). Though time-intensive, discourse analysis allows for unique insights into instructional communication in the visual, auditory, and tactile world of the operating room; this approach provides a much richer understanding than a traditional focus on purely oral instruction. Discourse analysis may similarly elucidate instructional techniques in other procedural settings from the endoscopy suite to the intensive care unit.

There are multiple limitations to note. We conducted this study with four instructors during simulation sessions, and differences in instruction may have related in part to instructors’ styles. All instructors were from a single large academic institution. Findings are likely to be most relevant to similar settings. Additionally, as we used a standardized learner to reduce learner variability, it was not possible from analysis of transcripts to determine whether learners would actually perceive ambiguity at all points that we identified as ambiguous. We defined referential ambiguity based on semantic ambiguities and lack of multimodality. However, it is likely that perceptions of ambiguity would depend on level of training. Furthermore, we looked at only one aspect of discourse – ambiguity – which represents a small part of instruction.

Future work will focus on analyzing instruction in unscripted settings with varied levels of learners, detailing other aspects of instruction beyond ambiguity that may affect learning, and better understanding the interplay between visual and oral instruction. First, given the importance of context to communication, analysis of unscripted settings with varied learners would provide a more nuanced understanding of experiences with instruction. Some learners may tolerate more ambiguity than others based on their prior knowledge. The diverse identities of learners and instructors, which have been previously suggested to affect post-operative communication, represent another area in which to study the manifestations of unscripted discourse during operations (Gates et al., 2023). Second, it would be useful to better understand other communication tools that facilitate instruction; for example, we speculate that measured repetition may help scaffold motor action. Finally, another direction could include determining whether combining visual and oral instruction together is *more* effective than precise oral instruction alone, as may be reasoned based on the cognitive theory of multimedia learning (Mayer, 2005). For example, while “move two centimeters anteriorly and one centimeter to the patient’s left” is precise, such a phrase could be more mentally taxing for learners to understand (and instructors to articulate) than a simply drawn arrow indicating the direction in which to move. Nonetheless, multimodality likely needs to be appropriately timed and placed to best provide clarity. Further work can delineate *how* best to harness multimodality to reduce cognitive load for all involved. These future directions will elucidate best instructional practices in the robotic operating room.

Overall, this study described, examined, and compared ambiguous instruction in remote and in-person simulated robotic surgery. Based on the high prevalence of ambiguity in both settings, we recommend that remote and in-person instructors work to reduce referential ambiguity. To do so, instructors can reduce semantic ambiguity, harness multimodality, or both.

## Data Availability

Selections of the data are provided within the manuscript and additional transcript data are available upon request.
